# The relationship between perception and landscape characteristics of recreational places with human mental well-being

**DOI:** 10.1038/s41598-025-88414-5

**Published:** 2025-02-04

**Authors:** Janina Vanhöfen, Talia Härtel, Giovanna Reichert, Christoph Randler

**Affiliations:** Didactics of Biology, Department of Biology, Eberhard Karls University Tübingen, Auf der Morgenstelle 24, 72076 Tuebingen, Germany

**Keywords:** Recreational places, Perceived naturalness, Landscape characteristics, Mental well-being, Place attachment, Biodiversity, Biodiversity, Human behaviour, Quality of life

## Abstract

Being outdoors benefits both humans’ physical and mental well-being, but the reasons for this connection are difficult to pinpoint. This study examines 40 recreational areas to understand their individual characteristics’ impact on mental well-being. We interviewed 1184 visitors on their perception of the place and feeling of mental well-being. In a second survey, 49 participants were shown images of the areas to assess whether they perceived them in the same way as people on-site did. Biologists then evaluated the areas through bird and biotope surveys, also assessing various other landscape characteristics of the study areas. Analysis using a two-level multivariate GLM revealed that perceived naturalness and perceived bird diversity are significant predictors of improved mental well-being. On the flip side, mental well-being declines with increased human impact, as indicated by the amount of infrastructure and the Human Footprint Index value. Notably, perceived naturalness positively correlates with actual bird diversity (*p* = 0.005) and negatively with human impact (*p* = 0.006). A strong perception of birds enhances the mental well-being of greenspace visitors (*p* < 0.001), while a high birding specialization negatively affects recalled restoration (*p* = 0.014). The estimated restoration value (*p* = 0.001) and estimated beauty (*p* < 0.001) from the online survey correlate positively with emotions experienced on-site. The perception of naturalness in images aligns with on-site perception, suggesting pathways for future research and the design of recreational areas.

**Supplementary Information:**

The online version contains supplementary material available at 10.1038/s41598-025-88414-5.

## Introduction

Mental health and well-being are an important part of human life. According to the Constitution of the World Health Organization (WHO), health “*is a state of complete physical, mental and social well-being and not merely the absence of disease or infirmity*”^1^. Health is strongly related to quality of life and therefore to areas frequented and lived in by people. Another important part of human life is the living situation. By 2030 approximately 60% of humanity will live in towns or cities^2^. In 2020 77.5% of Germans lived in cities, a rising trend^3^. This urbanization is not only a challenge for quality of life, but also one of the presumed leading causes for biodiversity loss^4^. Biodiversity, being the variety of life on earth, provides not only environmental services like air purification, temperature regulation and groundwater recharge, it’s loss is also a challenge to a healthier lifestyle of people^5^. In the face of biodiversity loss and climate change, the relationship between human health, mental well-being, and biodiversity is a rapidly expanding field of research^6^.

### The importance of biodiversity on mental well-being

Recently, evidence has emerged that biodiversity may support (short-term) well-being, possibly through the provision of ecosystem services^7^. Or by exposure to pleasant surroundings, or by encouraging health-promoting behaviours^8^. “Well-being” is a very broad term, which can be further diverted into physical, mental, and social well-being^9^. In the present study, we focus on mental well-being. According to Linton et al.^9^, mental well-being is connected to the psychological, cognitive and emotional quality of a person’s life, their thoughts and feelings about the state of their life and their experience of happiness. As it is an important aspect of human health, it is important to understand its predictors. In earlier studies, there even seemed to be an association between the presence of biodiversity and mental well-being^6,10,11^. For example, the experience of urban green spaces has increasingly been associated with greater mental well-being^12^ and lower levels of stress, depression and anxiety^13^ of people. People also smile more in parks with diverse shrubs and herbs^14^.

There are two important theory frameworks on the underlying mechanisms connecting natural environments to human mental well-being: attention restoration and stress reduction. The attention restoration theory (ART) by Kaplan and Kaplan^15^ indicates that spending time in nature allows recovery from attentional fatigue and restores the capacity to direct attention^16^. The stress reduction theory (SRT) suggests that natural environments benefit psychophysiological stress recovery due to people’s evolutionary connection to nature^17,18^.

Going further, Marselle et al.^19^ described pathways by which biodiversity is possibly linked to human mental well-being. One of them is by restoring capacities, adding the biodiversity dimension to the SRT and ART mentioned above. However, earlier research on the benefits of nature on human mental well-being often lacks specifics on ecological characteristics^20^ and the specifics of the biodiversity involved^21^. Regularly there is a focus on either flora or fauna, often missing the connection to landscape characteristics. Vegetational cover in urban areas has been positively associated with a lower prevalence of depression, anxiety and stress^22^. Generally, higher plant diversity in green spaces has been related to better mental well-being^11,19^. More varied and species-rich vegetation is also generally seen as aesthetically pleasing^23^ even though it does not always directly translate into preference^24^. Plant species richness may additionally aid in stress recovery^25^ and improve mental health^26^. Other studies focused on animal-related biodiversity. For example, many studies on biodiversity and well-being used bird species as an easily experienceable part of nature (see for example^27–30^), caused by birds being easily observed due to their behavioural characteristics^31^. In addition, people even seem to generally be happier in more wildlife-rich environments^27^.

But, beyond these simplifications of biodiversity, there is more to “nature” than just numbers of plants and animal species, namely the experience and perception of it. Every person is different and even though the ability to estimate the bird species richness seems to be roughly in line with actual bird species richness^32^, species knowledge and perception are often inaccurate^33^. Knowledge of bird species has also not been related to self-reported well-being in Chilean parks^34^. Hence, for experiencing nature, biodiversity and its benefits for mental well-being, accurate species knowledge is possibly not of high importance^35^. We aim to deepen the understanding of the effects of actual and perceived biodiversity of vegetation and animals (namely birds) on the mental well-being of visitors of recreational areas.

### The importance of recreational spaces for restoration and place attachment

People often develop emotional and cognitive bonds with a particular place they like—so-called “place attachment”^36–41^. The constructs of place attachment and place identity are strongly linked to the perceived restorativeness^42^ and the perceived restorative potential of an area^43^. Young et al.^44^ reported a greater connectedness of people to nature and greater restoration when visiting areas with higher environmental quality. Areas with a higher number of bird species nearby are unconsciously more satisfying to live in^45^. Animal diversity and nature-relatedness are also positively linked to perceived restorativeness which in turn increases positive affect and decreases negative affect^46^. Additionally, perceived restorativeness mediates the relationship between actual biodiversity and self-reported psychological and physical benefits, with participants in high biodiversity environments perceiving their environment as more restorative than those in low biodiversity environments^47^. Landscapes were also perceived as more natural and with higher restorativeness whenever they were objectively more diverse and natural^48^, with bird species richness influencing the perception of naturalness^49^.

### Landscape characteristics

Interaction with nature for urban dwellers mostly happens in recreational areas. These are areas or spaces of differing nature often in the city, but also in rural areas, which are intended and designed for recreational use, like parks. Often, the terms recreational areas and green spaces are used interchangeably since a lot of parks are mostly dominated by vegetation (“green”). According to the World Health Organization^50^ “greenspaces” are defined as “land covered by vegetation of any kind”. However, recreational areas are not limited to green spaces. A lot of recreational areas inside and outside of cities include bodies of water of differing sizes. If the body of water dominates the area it is generally called a “blue” space. These can be just as beneficial, perhaps even more so, than green spaces for humans^51^. While green spaces mainly include urban parks, forests, and gardens^44^, blue spaces include the ocean, rivers, lakes etc.^51,52^. Their recreational use has been shown to improve mood and attention, reducing depression, stress and anxiety^18,22,53–59^. Restoration in blue spaces was mostly related positively to landscape characteristics like beautiful views, bodies of water and greenery, but also to perceived restorativeness^60^. Still, the comparison between blue and green spaces and their connection to the effects of biodiversity on mental well-being is lacking in the literature.

Even though biodiversity as a concept is hard to grasp for a lot of people, naturalness being the proportion of anthropic versus natural elements is an easily understood concept^49^. Recreational areas are not only different in their amount of blue and green space but also in the physical elements present, like the amount of man-made structures or infrastructure. Human impact on the environment is ubiquitous. The Human Footprint Index (HFI), first published in 2022 and further developed by Venter et al.^61^ shows human impact at a spatial resolution of ~ 1 km. It uses data inputs on built environments, population density, electric power infrastructure, crop lands, pasture lands, roads, railways, and navigable waterways. To measure human pressure on the environment, remotely sensed and bottom-up surveyed information was compiled. The HFI is therefore a quantitative analysis of human influence across the globe. Human impact and other landscape characteristics may not only influence actual biodiversity in the areas^62^ but also the perception and experience of people existing in it.

### Goal of the current study

It remains unclear how recreational areas with their differing landscape characteristics connect to the mental well-being and nature experience of people using these areas for recreational purposes.

Therefore, we bring together many different aspects of earlier research to be able to paint a broader picture:

- Two different actual biodiversity measures (bird diversity^26,29,35,63^ and vegetational biotope types^35,64^),
- different landscape characteristics^65–67^,
- experience/perception of biodiversity and naturalness on-site^29,68^, and finally, we compare these to the
- perception of biodiversity/naturalness by pictures^48^.

**Fig. 1:**
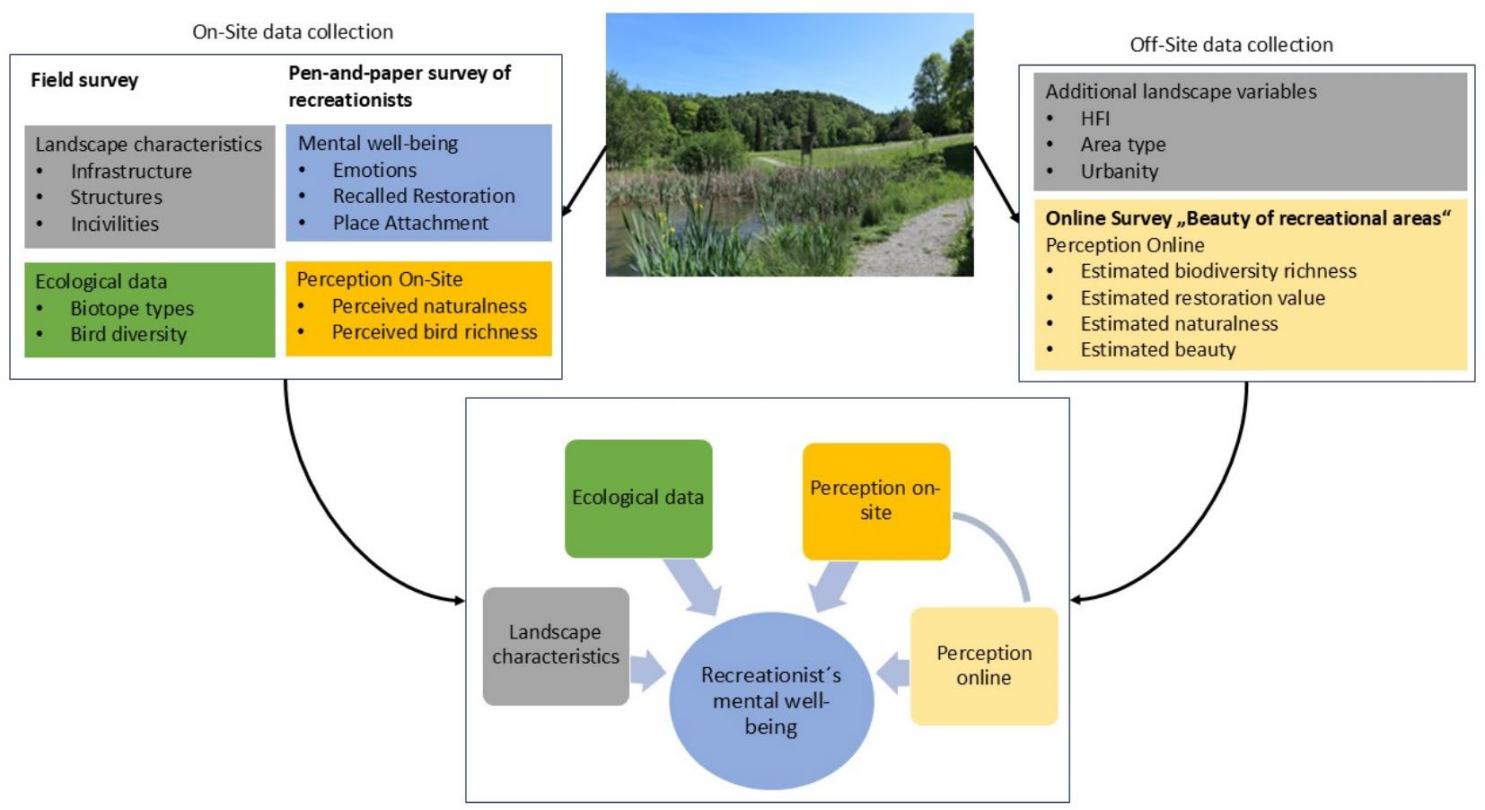
Scheme of research. 40 study locations in southwestern Germany were assessed on-site with a field survey and a pen-and-paper survey of recreationists. Additionally, off-site data collection regarding further landscape characteristics and online assessment of the areas was done. Variables were used to assess their connection with recreationists’ mental well-being, as well as the relationship between perception on-site and perception online.

To achieve this, we examined 40 recreational areas in southwestern Germany regarding their landscape characteristics and their effect on human mental well-being. In detail, we carried out three different surveys: (a) an ecological field survey done by biologists, (b) an on-site survey with recreationists, and finally (c) an online study to gather additional assessment (Fig. 1). Since recreational areas are not all built the same, their use and value to people differ tremendously. We would therefore expect different effects on people’s mental well-being depending on the area characteristics.

More precisely, we had the following research questions (RQs) and aims in mind:

1. How do the landscape characteristics affect the user’s mental well-being?
2. Does perceived bird diversity and/or naturalness on-site influence mental well-being?
3. Do participants assessing the restorative quality of recreational places via images perceive places similarly to people on-site?

## Materials and methods

To answer our research questions, we collected a variety of area characteristics and did two different surveys on users of recreational areas.

### Study locations

**Fig. 2:**
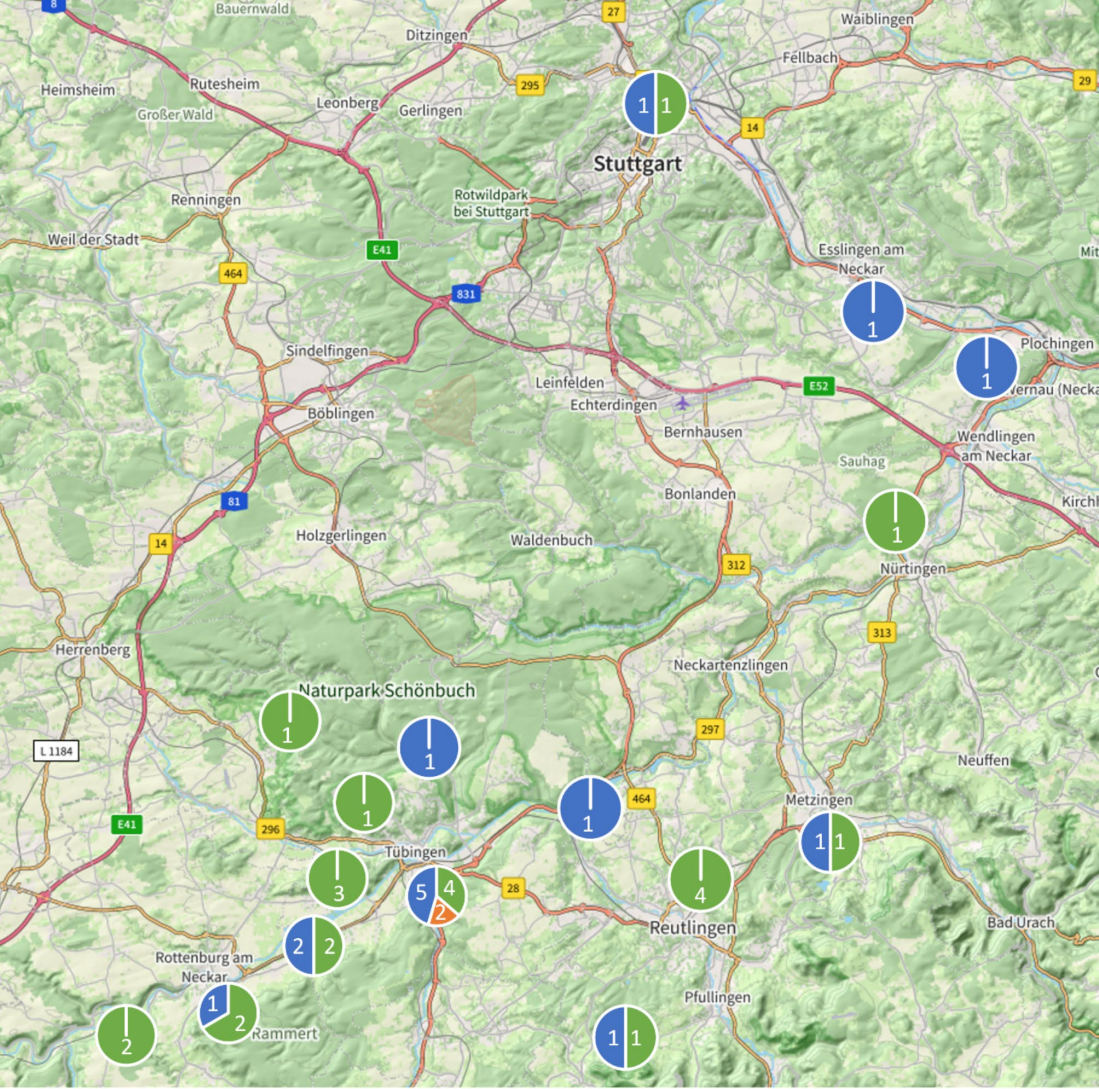
Map of the communes in which the study sites are located. Starting in the north with the city of Stuttgart, Baden-Württemberg, southwest Germany, going south along the Neckar River until just behind Rottenburg am Neckar. Circles marking the study areas are divided by their portion of present area types: blue spaces in blue, green spaces in green and mixed spaces in orange. Map data is copyrighted by OpenStreetMap contributors and available from https://www.openstreetmap.org.

Two surveys were carried out in 40 locations between Rottenburg am Neckar and Stuttgart along the Neckar River in Baden-Württemberg, a federal state in southwestern Germany. First, a field survey was conducted by biologists to assess ecological data. Second, recreationists were surveyed on their experience and mental well-being via a pen-and-paper questionnaire. Surveys were carried out in May and June of 2022 on days with fair weather. The sites vary in their degree of urbanization and include both green and blue spaces. All the sites are recreational areas easily accessible to people. These study sites were selected due to their popularity as recreational areas and there being easily accessible either by foot, bike, public transportation or car. See Fig. 2 for a map of the locations of the study sites.

### Area perception pen- and paper survey “biodiversity and well-being”

Pedestrians over the age of 18 were invited to participate in the questionnaire if they already had been in the respective area for a while. Participants were given the questionnaire on paper with a ballpoint pen. They were informed that it was anonymous, voluntary, and that they could stop at any time without consequences. This study has been granted ethical permission by the ethics committee of the Faculty of Social Sciences and Economics of the University of Tübingen (AZ: A2.5.4-219_ns) and has been carried out following all relevant guidelines and regulations. Informed consent was obtained from all participants.

#### Questionnaire

The questionnaire included 24 questions about location, perceived bird diversity, and well-being^32^. Demographic variables were age, gender, and the highest level of education. In addition, participants were asked to rate their ornithological skill level (*birding specialization* from Lee and Scott^69^, Randler and Heil^70^, and Randler^71^) and their *perception of birds*^72^.

We used three **outcome measures** for assessing mental well-being: *recalled restoration*, *emotions* and *place attachment*.

*Recalled restoration* after the visit was asked with one item, on a categorical scale with 6 categories (“significantly more recovered”, “somewhat recovered”, “about the same”, “less recovered”, “significantly less recovered”, “cannot say”), adapted from Young et al.^44^. For better comparability between the scales in our study, we recoded the recalled restoration Scale into a 0 (“cannot say”) to a 5 (“significantly more recovered”) Likert scale, with 0 being considered as missing data.

*Emotions* or self-reported restoration were measured with three items on a 5-point Likert scale (1 = fully disagree to 5 = fully agree), adapted from White et al.^51^ and Wyles et al.^52^. The scale had a Cronbach’s alpha of 0.77 in our study.

*Place attachment* (full scale with a Cronbach’s alpha of 0.88) can be split into two variables after a factor analysis, namely *place attachment-identity* and *place attachment-recovery*. The recovery scale is derived from the therapeutic value by the place attachment assessment of Bryce et al.^37^ and has a Cronbach’s alpha of 0.86 in our study. The identity scale is derived from the abbreviated three-item place identity scale of Boley et al.^36^ and has a Cronbach’s alpha of 0.87 in our study. Both scales use three items, coded on a Likert scale from 1 (“does not apply at all”) to 5 (“completely true”).

Additional variables asked for in the questionnaire were: *perceived bird diversity* at the respective places, asked on a categorical scale with nine categories (0–5; 6–10; 11–20; 21–30; 31–40; 41–50; 51–60; 61–70; over 70 species present; adapted after Ferraro et al.^73^ and Southon et al.^74^) and *perceived naturalness*, asked on a scale from 1 (unnatural) to 5 (very natural), adapted from Schebella et al.^65^. For a detailed description of the constructs and items used in the questionaire, please see Table A1 in the Appendix.

### Biodiversity data collection

In a field survey, accompanying the pen-and paper survey, biologists collected data on actual biodiversity present in the study locations.

#### Bird diversity

Actual bird species richness on site was recorded by trained ornithologists using standard protocols^75^. In a 250 m radius around footpaths which visitors experience, all bird species seen and heard in 15 min were noted. Data was collected between March and June 2022, based on three visits at least 4 weeks apart. Here, we used the total bird count of all recorded bird species (see also Dallimer et al.^29^ and Vanhöfen et al.^32^). This list therefore includes all bird species encountered at one location so that each species contributed to the dataset only once.

#### Biotope types

Several previous studies tried to assess and categorize areas. Voigt et al.^67^ proposed the three dimensions of the *biotic*, *abiotic*, and *man-made* to assess urban parks. In a further development, Gidlow et al.^66^ developed the natural environment scoring tool, which comprises 47 items in 8 domains: accessibility, recreational facilities, amenities, aesthetics-natural, aesthetics-non-natural, significant natural features, incivilities and usability. This list is comprehensive, but not all domains apply to our study areas. In their assessment, Schebella et al.^65^ used checklists examining structural elements, habitat types and anthropic elements. They also proposed an urban park naturalness index including biotic elements, artificial elements, input and extraction, dynamics, and general human dependence.

Both tools are not easily applicable to our study areas without modification. Therefore, we adapted the ideas of all three mentioned authors to an area survey more easily applicable to our study areas by trained biologists (for a detailed description see Table A2 in the Appendix).

The biotic vegetational composition and structure of the areas were recorded using the determining key catalogue “Arten, Biotope, Landschaft” from the nature conservation administration Baden-Württemberg^76^ which serves as a uniform reference system for biological data collection projects. Using this key, biologists noted all biotope types (out of 64 possible Items) in the respective areas, using the same tours and roads normal visitors would use. The resulting variable *biotope types* includes the number of all different biotope types present in the respective areas, representing the richness of possible habitats for biodiversity and especially the species richness of plants.

### Landscape characteristics

Different landscape characteristics were collected, either directly on the study sites or in a second step using map data.

#### On-Site landscape variables

To assess the abiotic elements, the biologists conducting the field survey also noted all man-made elements present in the areas (see also Table A3 in the Appendix for further information). From this, reasonable categories in adherence to Gidlow et al.^66^ and Schebella et al.^49,65^ were built. *Structures* (like buildings, roads, bridges, etc.) included the sum of most man-made structures not fitting in with the variable of *infrastructure*. *Infrastructure* included the sum of factors like accessibility, parking places, hiking routes, signage, benches toilets, lighting, etc. *Incivilities* included trash and vandalism, among other things.

#### Off-Site landscape variables

##### Urbanity

As a further assessment of the area characteristics, we dichotomised areas as either rural or urban to get the variable of *urbanity*. Since most of Germany is very densely populated, this definition is mostly a subjective assessment, influenced by present structures. Areas were defined as rural if they were mostly situated in open farmland, grassland, marsh, or woodland, containing only a few elements like roads, large buildings, and houses^77^. Urban areas in contrast contained a matrix of built-in areas, like roads, houses, or large buildings. The urban recreational areas within this matrix still contained nature-based features such as gardens, parks, or water bodies. According to Reichert^77^, rural sites were coded with a 2, and urban sites were coded with a 1.

##### Blue and green spaces—area type

To assess further the characteristics of the areas of interest, we also measured the percentage of blue spaces and green space using QGIS (3.32.1) and the QuickOSM plugin (2.2.3). Since the federal state of Baden-Württemberg does not participate in the geodata offered by the federal government of Germany, the official database of the federal government uses the Open Street Map Data of Baden-Württemberg, making it sufficient for our use^78^ (Bundesamt für Kartographie und Geodäsie, 2023). The Open Street Map database is distributed under the Open Database Licence (ODbL) 1.0 and is available under https://www.openstreetmap.org/ (last assessed on 05.10.2023).

Using these percentages, places were then further categorized regarding their features as a blue-, green- or mixed area, creating the variable *area type*.

Since blue spaces are generally outdoor environments with a prominent water feature and accessible to humans either proximally or distally^79^, we used the percentage of waterbodies in an area to define them as follows: blue space is composed of at least > 25% water body and is coded as 1, green space is composed of > 50% vegetational area and is coded as 2. If neither or both of these apply, the area is labelled as mixed and coded as 3. As our study sites are roughly located along the Neckar River, spaces categorized as blue mainly include the river, large waterbeds, and different forms of large ponds. At the same time, mixed spaces include a high percentage of green space with only small water streams and bodies. As green categorized spaces do not contain much water but cover multiple vegetation types, like woodland, grassland, parks, and gardens. Of the 40 study sites, 37.5% are categorized as blue space, 57.5% as green space and 5% as mixed space (meaning neither blue nor green components predominate). 47.5% are urban and 52.5% are rural areas.

##### Human footprint index

The Human Footprint Index (*HFI*), developed by Venter et al.^61^ shows the human impact on a respective area. Pressures are scaled between 0 and 10 and afterwards weighted according to estimates of their relative level and summed together to create the standardized HFI. For each 1 km^2^ the HFI can range from 0 to 5, with a lower score indicating less human impact. We assessed this data last on 10.08.2023^80^ by using QGIS (Version 3.32.1).

### Area perception online-study “beauty of recreational areas”

In a separate but related online survey, the 40 areas used for the on-site assessment were randomly presented to an independent sample of respondents via an online survey. To ensure the quality of responses, the participants were asked to complete the survey task in the presence of working group members. Participants received a book for compensation to increase compliance.

This survey was distributed within the student body of a large German university and was hosted on the German SoSci-Survey server to comply with the European Union’s data privacy rules.

Demographic variables asked for were gender, age, and highest level of education, to check for bias in participation. The demographic results were not used directly in this analysis on place level.

**Fig. 3:**
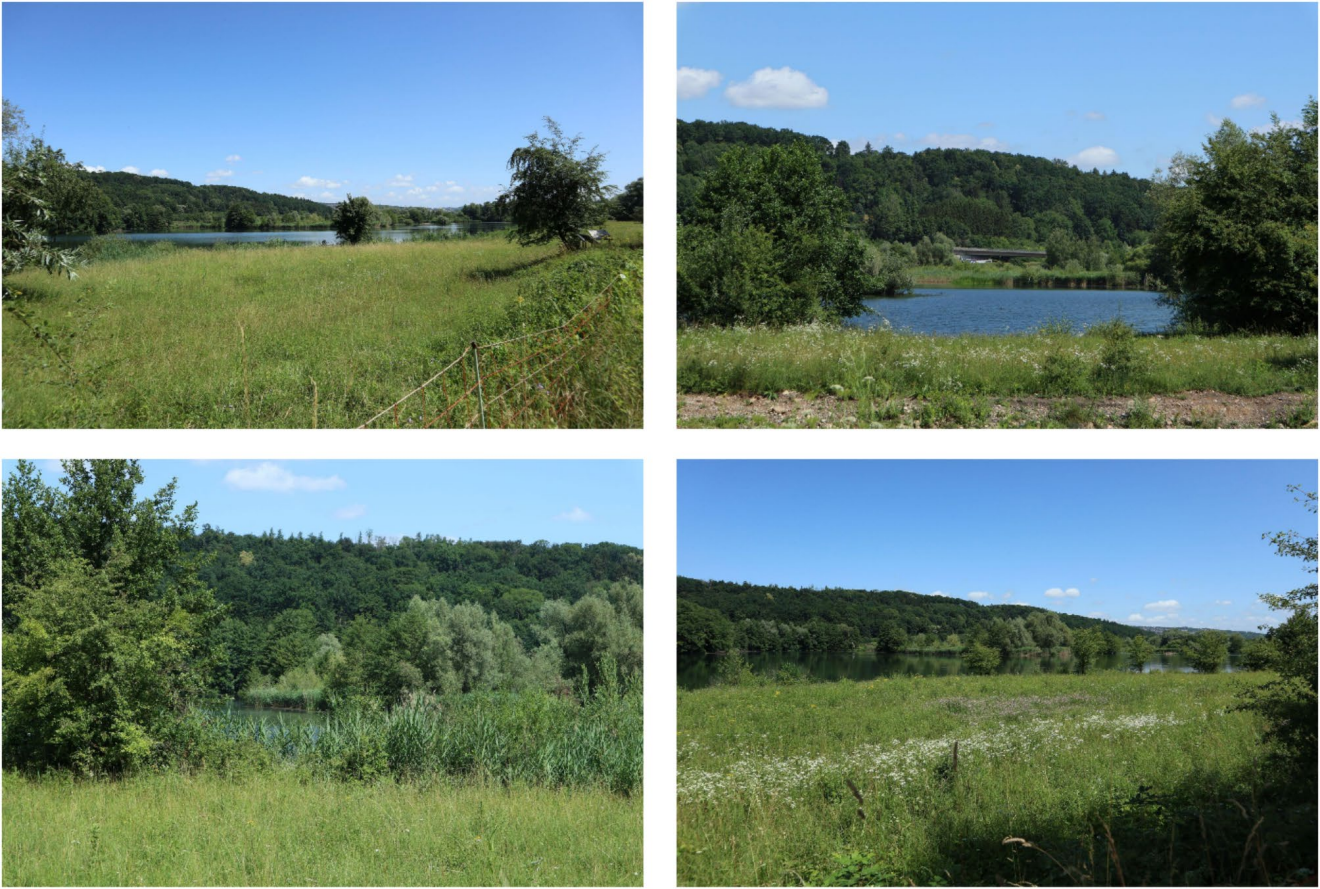
Wernau dredging lake, example for a rural blue space—as shown to participants of the area-perception online study “Beauty of recreational areas”.

Participants were shown images of the same 40 places where the on-site survey took place (photographed by authors JV and TH, Fig. 3 shows one example presented to participants). Four pictures were shown per site. For each study area, we asked for the participants’ assessment of (see full survey catalogue in Table A4 in the Apendix):

- *Estimated beauty* of the area on a scale from 1 (not at all beautiful) to 5 (very beautiful)
- *Estimated the naturalness* of the area on a scale from 1 (unnatural) to 5 (very natural) which is the same question as in the “biodiversity and well-being” survey and derived from Schebella et al.^49,65^
- *Estimated biodiversity richness* on a scale from 1 (low) to 5 (high), derived and adapted from White et al.^81^.
- *Estimated restoration value* on a scale from 1 (none) to 5 (very high)^81^.

### Statistical analysis

We did our analysis two ways: (1) on the place level, following previous studies^29,49,82^, using the means per place calculated from the individual surveys. And (2) in a hierarchical structure, using individual-level data. The program SPSS (Version 29.0.1.1) was used for all statistical analyses.

Before analysing the research questions, the descriptive statistics of predictor variables and study areas were assessed at the place level. To check if the assessments of biologists and participants are related to the variables of area type and the HFI, we carried out a non-parametric Spearman-rho correlation on the landscape characteristics.

To analyse the research questions 1 and 2, we did a multivariate two-level GLM on the outcome variables (mental well-being: emotions, recalled restoration, place attachment-identity and place attachment—recovery) with the individual data points (N = 1184) as level 1 the place-level (N = 40) as superior level 2. The same model is used to see the effects of landscape characteristics on visitors’ mental well-being (RQ1), and if perception on-site (and online) influences the mental well-being of visitors (RQ2). To be able to determine the effect direction, an additional Pearson correlation (*p*-values Bonferroni–Holm corrected) as well as a graphical inspection was done. For RQ2, a nonparametric Spearman-rho correlation of the perception on-site and the perception online with the landscape characteristics was done to analyse a possible connection between landscape characteristics and the perception.

After, we did a Spearman-rho correlation on the online survey “Beauty of recreational areas” to see if the perception while looking at images during the online study is in line with the results of the pen and paper study “biodiversity and well-being” (RQ3). We checked if participants online had the same outcome in perceived bird diversity and perceived naturalness and wanted to see if this correlated with bird diversity and biotope types. Since two of our predicting variables, area type and urbanity, are nominally coded, we did a Chi^2^ to analyse their relationship with the other variables.

In all correlation analyses the *p*-values were Bonferroni–Holm corrected.

## Results

### Descriptive statistics

The area perception on-site survey “biodiversity and well-being” had 1184 participants. There were 30 to 31 participants per area, except for two study sites (N = 17 and 24). 54.9% of the participants were female, 44.1% were male (0.2% identified as diverse, 0.8% did not answer the question). A university degree (at least on the bachelor’s level) was reported by 55.7% of the participants. Participants` ages ranged from 18 to 89 years old (1.9% did not answer) with the mean age being 43.34 ± 17.75 years.

The area perception online study “beauty of recreational areas” had 49 participants with a surplus of females (34.7% male, 63,3% female, and 2% did not answer the question). The mean age was 32.69 ± 13.63 years (range 19–67). About half of the participants did not have a university degree (55.1%): 16.3% had a secondary school diploma (“Realschulabschluss”), 38.8% had a high school diploma (“Abitur”), meanwhile 40.8% reported some university degree (4% did not answer or selected “different”).

Table 1 shows the descriptive statistics of the study areas, depending on the respective area type. The descriptive statistics of all variables are presented in Table A5 in the Appendix.

**Table 1:** Descriptive statistical information on the study areas, dependent on their area type (blue/green/mixed space) (N = 40).

	Area type	Mean	Std. deviation	Minimum	Maximum	Range
HFI	Blue	35.989 ± 0.396	8.291	13.0	45.0	32.0
	Green	32.239 ± 0.410	10.737	14.0	46.0	32.0
	Mixed	30.000 ± 0.260	2.017	28.0	32.0	4.0
Urbanity	Blue	1.450 ± 0.024	0.498	1.0	2.0	1.0
	Green	1.606 ± 0.019	0.490	1.0	2.0	1.0
	Mixed	1.000 ± 0.000	0.000	1.0	1.0	0.0
Biotope types	Blue	12.547 ± 0.101	2.114	9.0	16.0	7.0
	Green	13.568 ± 0.170	4.449	7.0	22.5	15.5
	Mixed	16.500 ± 0.456	3.530	13.0	20.0	7.0
Structures	Blue	5.521 ± 0.068	1.427	3.0	8.0	5.0
	Green	5.305 ± 0.075	1.957	2.0	10.0	8.0
	Mixed	8.000 ± 0.000	0.000	8.0	8.0	0.0
Infrastructure	Blue	5.487 ± 0.104	2.181	1.5	10.5	9.0
	Green	7.203 ± 0.072	1.887	4.5	11.0	6.5
	Mixed	6.250 ± 0.033	0.252	6.0	6.5	0.5
Incivilities	Blue	1.726 ± 0.041	0.865	0.0	3.0	3.0
	Green	0.920 ± 0.032	0.830	0.0	3.0	3.0
	Mixed	3.000 ± 0.391	3.025	0.0	6.0	6.0
Bird diversity	Blue	29.030 ± 0.251	5.247	22.0	42.0	20.0
	Green	28.250 ± 0.249	6.513	18.0	41.0	23.0
	Mixed	23.500 ± 0.195	1.513	22.0	25.0	3.0

**Fig. 4:**
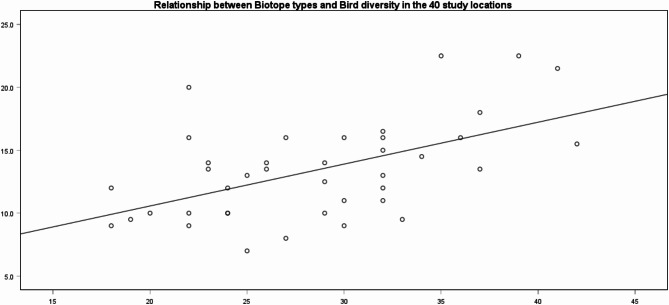
Point-plot of the actual biodiversity, in the 40 study areas. The relationship between biotope types and bird diversity is shown with a regression line (R^2^ linear = 0.292).

Looking at the landscape characteristics, the biotope types correlate with bird diversity (rho = 0.496, *p* = 0.006) and HFI (rho = − 0.477, *p* = 0.012). The bird diversity additionally correlates negatively with the HFI (rho = − 0.472, *p* = 0.012). This confirms that in areas with a high number of biotope types there are more bird species with the opposite being true for areas with a high human impact factor. See Fig. 4 for a presentation of the distribution of actual biodiversity in the 40 study areas. It also confirms the wide variety of recreational areas we used.

In addition, area type is related to incivilities (*X*^2^ (8, *N* = 40) = 31.179, *p* < *0.001*), and urbanity is related to the HFI (*X*^2^ (20, *N* = 40) = 34.319, *p* = 0.024). These results are probably related to the usage of the areas, a topic outside the scope of this manuscript. Urbanity being connected to the HFI confirms that more urban areas have a higher HFI. The structures and infrastructure variables do not correlate with any of the other area characteristics assessed.

### Effects of landscape characteristics on mental well-being (RQ1)

Mental well-being was measured with the variables recalled restoration, emotions, place attachment-identity, and place attachment-recovery.

The two-level GLM (Table 2) shows significant effects of gender, birding specialization, age, HFI, and infrastructure on the mental well-being variables.

**Table 2:** Hierarchical 2-level GLM, with individual-level data (N = 1184) as level 1 and study site (N = 40) as level 2, corrected for individual sociodemographic variables.

Source	Dependent variable	Numerator df	Denominator df	F	Sig
Intercept	**Emotions**	**1**	**1049**	**56.929**	** < 0.001**
	**Recalled restoration**	**1**	**1044**	**19.144**	** < 0.001**
	**Place attachment—identity**	**1**	**1050**	**23.890**	** < 0.001**
	**Place attachment—recovery**	**1**	**1051**	**34.738**	** < 0.001**
Area type	Emotions	2	1049	0.388	0.678
	Recalled restoration	2	1044	0.532	0.588
	Place attachment—identity	2	1050	0.632	0.532
	Place attachment—recovery	2	1051	0.738	0.478
Urbanity	Emotions	1	1049	0.038	0.845
	Recalled restoration	1	1044	0.622	0.430
	Place attachment—identity	1	1050	2.821	0.093
	Place attachment—recovery	1	1051	0.974	0.324
Gender	Emotions	1	1049	1.277	0.259
	**Recalled restoration**	**1**	**1044**	**4.389**	**0.036**
	Place attachment—identity	1	1050	0.164	0.686
	**Place attachment—recovery**	**1**	**1051**	**7.032**	**0.008**
Graduation	Emotions	1	1049	0.089	0.766
	Recalled restoration	1	1044	0.440	0.508
	Place attachment—identity	1	1050	0.015	0.903
	Place attachment—recovery	1	1051	0.002	0.968
Birding specialization	Emotions	5	1049	0.741	0.593
	**Recalled restoration**	**5**	**1044**	**2.853**	**0.014**
	Place attachment—identity	5	1050	1.316	0.255
	Place attachment—recovery	5	1051	1.066	0.377
Age	Emotions	1	1049	0.261	0.610
	**Recalled restoration**	**1**	**1044**	**5.964**	**0.015**
	**Place attachment—identity**	**1**	**1050**	**63.245**	** < 0.001**
	Place attachment—recovery	1	1051	0.027	0.870
HFI	**Emotions**	**1**	**1049**	**11.280**	** < 0.001**
	Recalled restoration	1	1044	1.754	0.186
	**Place attachment—identity**	**1**	**1050**	**4.830**	**0.028**
	Place attachment—recovery	1	1051	0.919	0.338
Estimated biodiversity richness	Emotions	1	1049	0.026	0.871
	Recalled restoration	1	1044	1.334	0.248
	Place attachment—identity	1	1050	1.475	0.225
	Place attachment—recovery	1	1051	0.002	0.963
Estimated restoration value	**Emotions**	**1**	**1049**	**10.857**	**0.001**
	Recalled restoration	1	1044	1.797	0.180
	Place attachment—identity	1	1050	0.017	0.897
	Place attachment—recovery	1	1051	0.589	0.443
Estimated naturalness	Emotions	1	1049	0.014	0.906
	Recalled restoration	1	1044	0.403	0.525
	Place attachment—identity	1	1050	3.031	0.082
	Place attachment—recovery	1	1051	1.331	0.249
Estimated beauty	**Emotions**	**1**	**1049**	**17.841**	** < 0.001**
	Recalled restoration	1	1044	1.354	0.245
	Place attachment—identity	1	1050	0.000	0.990
	Place attachment—recovery	1	1051	0.452	0.501
Biotope types	Emotions	1	1049	0.019	0.892
	Recalled restoration	1	1044	0.488	0.485
	Place attachment—identity	1	1050	0.157	0.692
	Place attachment—recovery	1	1051	0.084	0.772
Structures	Emotions	1	1049	1.687	0.194
	Recalled restoration	1	1044	0.022	0.883
	Place attachment—identity	1	1050	0.015	0.901
	Place attachment—recovery	1	1051	0.491	0.484
Infrastructure	Emotions	1	1049	0.148	0.700
	Recalled restoration	1	1044	1.979	0.160
	**Place attachment—identity**	**1**	**1050**	**22.971**	** < 0.001**
	**Place attachment—recovery**	**1**	**1051**	**4.803**	**0.029**
Incivilities	Emotions	1	1049	1.573	0.210
	Recalled restoration	1	1044	0.542	0.462
	Place attachment—identity	1	1050	0.374	0.541
	Place attachment—recovery	1	1051	0.341	0.560
Bird diversity	Emotions	1	1049	1.256	0.263
	Recalled restoration	1	1044	2.567	0.109
	Place attachment—identity	1	1050	0.091	0.762
	Place attachment—recovery	1	1051	0.092	0.762
Perceived bird diversity	Emotions	1	1049	1.737	0.188
	**Recalled restoration**	**1**	**1044**	**6.586**	**0.010**
	Place attachment—identity	1	1050	1.709	0.191
	Place attachment—recovery	1	1051	2.036	0.154
Perceived naturalness	**Emotions**	**1**	**1049**	**61.288**	** < 0.001**
	**Recalled restoration**	**1**	**1044**	**4.591**	**0.032**
	**Place attachment—identity**	**1**	**1050**	**18.370**	** < 0.001**
	**Place attachment—recovery**	**1**	**1051**	**33.438**	** < 0.001**
Perception of birds	**Emotions**	**1**	**1049**	**95.565**	** < 0.001**
	**Recalled restoration**	**1**	**1044**	**20.914**	** < 0.001**
	**Place attachment—identity**	**1**	**1050**	**68.720**	** < 0.001**
	**Place attachment—recovery**	**1**	**1051**	**60.693**	** < 0.001**

Significant results are written in bold letters.

The sociodemographic variables have various impacts on the mental well-being of participants. Female participants report higher recalled restoration and higher place attachment–recovery values than male participants. The older the participants the higher they rate their recalled restoration and place attachment—identity. Participants with higher self-reported ornithological skill rate their recalled restoration lower than participants with less birding specialization.

The landscape characteristics only impacted participants negatively: in places with higher HFI the emotions and place attachment-identity ratings were lower. Additionally, more infrastructure led to lower place attachment-identity and place attachment-recovery.

### Effects of perception on mental well-being (RQ2)

#### Landscape characteristics and perception

The perceived naturalness on-site correlates positively with bird diversity and negatively with the HFI (Table 3). Thus, people perceive places as more natural if they present a higher bird diversity with a small human impact.

**Table 3:** Nonparametric spearman-rho correlation of the perception on-site and the perception online with the landscape characteristics (N = 40).

	Perceived bird diversity		Perceived naturalness		Estimated biodiversity richness		Estimated restoration value		Estimated naturalness		Estimated beauty	
	rho	cor. p	rho	cor. p	rho	cor. p	rho	cor. p	rho	cor.p	rho	cor. p
Biotope types	0.333	0.186	0.347	0.168	0.449**	**0.024**	0.359	0.138	0.368	0.114	0.422*	**0.042**
Structures	− 0.231	0.456	− 0.310	0.306	− 0.100	> 0.999	− 0.123	> 0.999	− 0.198	> 0.999	− 0.033	> 0.999
Infrastructure	− 0.111	0.594	0.040	> 0.999	− 0.054	> 0.999	0.058	> 0.999	− 0.101	> 0.999	0.023	> 0.999
Incivilities	− 0.169	0.594	− 0.218	> 0.999	− 0.156	> 0.999	− 0.098	> 0.999	− 0.100	> 0.999	0.006	> 0.999
HFI	− 0.343	0.186	− 0.558**	**0.006**	− 0.678**	**0.006**	− 0.616**	**0.006**	− 0.592**	**0.006**	− 0.559**	**0.006**
Bird diversity	0.325	0.186	0.517**	**0.005**	0.492**	**0.006**	0.349	0.162	0.487**	**0.006**	0.403	0.06

*p* values were Bonferroni–Holm corrected. Significant values are written in bold letters.

The number of biotope types is correlated with the estimated biodiversity richness and estimated beauty of the area perception online study. The HFI, in addition to perceived naturalness, correlates negatively with all variables of the area perception online study. From this, we confer that people looking at images are negatively influenced in their perception of biodiversity, naturalness, beauty, and restoration value by a high human impact. Structures, infrastructure, and incivilities do not play a role since they do not correlate with the perception variables. Area type and urbanity are not related to any of the perception values according to the chi^2^ test we did.

Bird diversity additionally correlates with the estimated biodiversity richness and estimated naturalness of the online study. Therefore, if there are a lot of bird species present, people looking at areas per images perceive them as higher in biodiversity and naturalness.

#### Perception and mental well-being

The two-level GLM (Table 2) shows the effects of estimated restoration value, estimated beauty, perceived bird diversity, perceived naturalness and perception of birds on the mental well-being of participants.

Participants with a more positive perception of birds reported more positive emotions, higher recalled restoration, place attachment-identity and place attachment-recovery.

Places with higher perceived bird diversity led to higher recalled restoration ratings of participants.

**Fig. 5:**
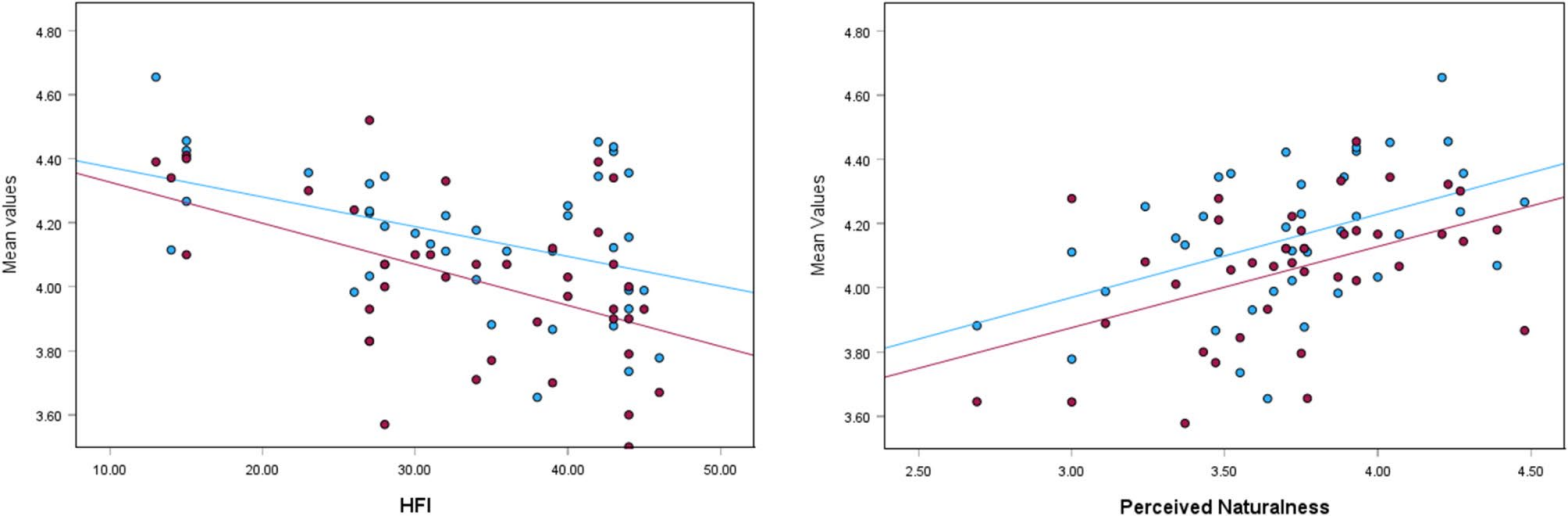
(Left) The negative relationship between the HFI and emotions respectively recalled restoration. Blue = emotions, red = recalled restoration. R^2^ linear (emotions, blue line) = 0.175, R^2^ linear (recalled restoration, red line) = 0.245). (Right) The positive relationship between place attachment—recovery, emotions and perceived naturalness. Blue = emotions, red = place attachment—recovery. R^2^ linear (both lines) = 0.215.

High perceived naturalness led to more positive emotions (Fig. 5 right), higher recalled restoration, place attachment-identity and place attachment-recovery. The HFI has a negative relationship with emotions and recalled restoration (Fig. 5 left).

The online perception of the areas is also connected to mental well-being on-site: higher estimated restoration value and estimated beauty online are connected to more positive emotions on-site.

### Perception on-site and online (RQ3)

All four variables for the area perception online study correlate with the perceived naturalness of a place (Table 4). In contrast, they are not related to the perceived bird diversity by people asked in the area. Therefore, if a place is perceived as natural by people on-site, people looking at images also find it to be more biodiverse, natural, beautiful and of a higher restoration value.

**Table 4:** Non-parametric spearman-rho correlations (N = 40) comparing the area perception online study with the area perception on-site survey.

	Perceived bird diversity			Perceived naturalness		
	rho	*p*	cor. p	rho	*p*	cor. p
Estimated biodiversity richness	0.378	0.016	0.056	0.610**	** < 0.001**	**0.004**
Estimated naturalness	0.386	0.014	0.056	0.607**	** < 0.001**	**0.004**
Estimated restoration value	0.21	0.194	0.38	0.534**	** < 0.001**	**0.004**
Estimated beauty	0.212	0.19	0.38	0.472**	**0.002**	**0.004**

Significant results are written in bold letters.

## Discussion

In this paper, we compare two different survey studies and use different area characteristics to assess the impact on visitors’ mental well-being in recreational areas.

Results of the GLM show that sociodemographics are also connected to the mental well-being of recreationists in the study areas. Female participants have a higher recalled restoration and higher place attachment-recovery than male participants. This is probably due to individual differences predisposing some females to higher restoration benefits from green space^83^.

In addition, older participants rate their recalled restoration and place attachment-identity higher than younger participants.

Additionally, participants with a high perception of birds reported more positive emotions, higher recalled restoration, place attachment-identity and place attachment-recovery. These are probably people with a stronger connection to nature benefitting from being outside. Connectedness to nature is the emotional connection to living animals and nature^84^. This emotional connectedness to nature can influence participation in nature-based activities^85^ and probably also does so in our study.

Interestingly, participants with higher ornithological skills rate their recalled restoration lower. These participants could have been on a birding trip or similar and might have been frustrated by not meeting their goals. In earlier studies, Randler et al.^40^ found that non-birders have higher satisfaction and restoration than specialized birders after a birding trip since the feeling of success arrives more quickly for them.

### How do the landscape characteristics affect the user’s mental well-being? (RQ1)

The different biotope types and the actual bird diversity were not connected to mental well-being variables according to the model (Table 2). Even though some large-scale survey studies like Methorst et al.^26^ found a significant positive relationship between bird species richness and mental health in Germany using panel surveys of private households, our result is in line with other on-site studies which found no relationship between mental well-being and actual bird species richness^22,29,86^. Bird species richness therefore seems to not improve mental well-being directly on-site, but still could have an indirect and/or delayed positive impact on people. Generally, having more species in a neighbourhood has been shown to have a positive effect on well-being^26,63,82,87^. In our study, it is not the actual bird diversity per se which impacts people’s mental well-being on-site. Rather, the experience in the area is the decisive factor—as discussed regarding the RQ2 and RQ3. Even though we did not find a direct connection between our biodiversity measures and mental well-being, it could be that places promoting high bird diversity have beneficial landscape properties, which also benefit people’s well-being^88^. This is probably partly due to aesthetic reasons^23^, since the number of different biotope types in an area does not affect visitors’ mental well-being according to our results. People may either differentiate or experience biotope types in terms of vegetation in general leading to stress reduction^22^. And more varied green spaces were related to better mental well-being in previous studies^11,19,26^. Or recreationists also could only find the “larger picture” of the landscape to be beneficial to their mental health^89^.

In addition to biodiversity, as recorded by biologists, we collected data on existing infrastructure, structures, and incivilities. A higher presence of infrastructure led to lower place attachment-identity and place attachment-recovery. Infrastructure in our study means connections, accessibility, car parks, hiking routes, signposting, benches, playgrounds, etc. These are mainly well-developed areas that are likely to be visited by many people. They may be perceived as noisier, deprived, and polluted. Earlier, perceived traffic was negatively related to restoration in a study by Subiza-Pérez et al.^60^.

In our study areas, incivilities such as trash or graffiti did not affect place attachment—recovery. Incivilities were only present with a mean score of 1.33 ± 1.21 out of 6, their rare presence had seemingly no impact on visitors. Structures on the other hand are present with a mean of 5.51 ± 1.83 out of 10. Structures in our study are man-made things like buildings, roads, bridges etc., which are necessary for access to the areas and a common sight for people. It follows that these are perceived as neutral, especially for urban dwellers, and do not have a direct strong impact on our assessments of mental well-being or place attachment.

We also measured urbanity, area type and the HFI of the areas and tried to relate them to the people’s mental well-being.

Area type and urbanity do not affect our outcome variables—it does not matter to people if an area is a green or blue space, the most important thing is that it is an outside or natural area^90^. It has to be kept in mind that most landscapes are not always one type or the other as most consist of many different characteristics which in turn can influence mental well-being and/or perception.

In study areas with a higher HFI, the emotions and place attachment—identity were lower than in areas with lower HFI (Table 2). From this we conclude that more important than the type of an area is the human impact present in it. Areas with greater human impact elicit more negative emotions and may even be harmful to restoration since restoration as a concept is seen as an evolutionary-based response to certain landscapes^15,18^.

Participants in our study felt more attached to areas with fewer buildings, infrastructure, and other developments. The HFI is negatively connected to mental well-being in our study, meaning that areas with a high human impact are not as beneficial to visitors´ mental health. People express more positive emotions in more rural places even though urbanity as a variable does not predict mental well-being. Perception of a place is therefore perhaps the most important predictor of mental well-being.

### Does perceived biodiversity and or naturalness on-site influence mental well-being? (RQ2)

High perceived naturalness led to more positive emotions, higher recalled restoration, place attachment—identity and place attachment- recovery, according to the model (Table 2). Additionally, a higher perceived bird diversity led to higher recalled restoration of participants.

The results also show that the estimated restoration value and estimated beauty when looking at pictures are also related to emotional and restorational outcomes on-site.

This adds to the growing evidence that it is the perception of a place that is important when experiencing it. Previous studies^11,29,82^ showed that perception of species richness is important for human mental well-being. However, we have also shown that the perceived naturalness of an area is important. As in the study by Schebella et al.^49^, the perceived measures of biodiversity are impacting well-being instead of actual/objective measures (e.g. actual bird diversity).

This matches the results mentioned in the first part of this discussion: areas with less infrastructure and human impact are perceived as more natural and therefore also affect mental well-being. Since the perceived naturalness of a place is negatively correlated with the HFI (Table 3), people can correctly associate areas with a high human impact as less “natural”. The definition of “nature” is not clear, but Hartig et al.^91^ defined it as “physical features and processes of non-human origin” which suits the general perception of the public seeing “natural” as the counterpart of “human”. However, due to the subjectivity of the definition, it is difficult to determine what people perceive as natural. Some management seems desirable, as sites left completely untouched are often perceived as less aesthetic than extensively managed sites^92^. For example, managed meadows have a more positive impact on stress reduction than abandoned meadows in the Alps^93^. Since people seem to perceive naturalness/biodiversity visually^94^, mostly concentrating on vegetation^68^ and especially noticing trees^95^, the personal definition of “natural” could be quite different and depend strongly on the socialization of participants. However, according to our findings, this perception of naturalness is of particular importance when it comes to promoting mental well-being in recreational areas. Wilson’s^96,97^ theory of biophilia states humans feel most at home and are best able to flourish in places that mimic the natural spaces in which the species evolved. More natural areas with less infrastructure and a lower HFI fit this description probably best for most people.

### Do participants assessing the restorative quality of recreational places via images perceive places similarly to people on-site? (RQ3)

In a related online survey, we showed participants pictures of the areas surveyed in the on-site study and asked for their perception of these places. The estimated biodiversity richness, estimated naturalness, estimated restoration value, and estimated beauty of this survey are correlated with the perceived naturalness of the on-site survey (Table 4). Therefore, people’s perceptions of areas are similar if not the same, no matter if asked on-site or shown pictures The estimated restoration value is additionally connected to emotions and recalled restoration on-site. The perception of an area as restorative could well be the subjective landscape characteristic important for improving mental well-being. The effect on the mental well-being of online participants was not tested in this study, but other studies found positive results^98^.

Areas perceived as more restorative are probably landscapes with greater naturalness and ecological diversity, as areas like this were assessed as more natural and positively associated with greater perceived restorativeness in the study by Chang et al.^48^. Areas with a greater presence of biotope types and higher bird diversity are perceived to be more biodiverse and beautiful (Table 3). Areas perceived to be more natural have higher bird diversity, just as the perceived naturalness of participants on-site. As already shown by Vanhöfen et al.^32^ people on-site can roughly estimate the bird diversity as recorded by biologists. People online were seemingly just as good at estimating species richness as the people on-site. Estimated biodiversity richness and estimated beauty are also related to the number of biotope types. This strengthens the idea that people estimate biodiversity independently of their experience of it. However, it is unknown which variables and impressions lead to the assessment of a biodiversity-rich area. This could be studied further using qualitative interviews.

Additionally, the HFI was related to perceived naturalness on-site as well as estimated biodiversity richness, naturalness, restoration value and beauty, giving further evidence that people can perceive a place similarly, regardless of experience. They do not need to be there physically to estimate naturalness and species richness. This also means that people use other measures than experiencing a species or biodiversity itself in their evaluation of a place.

## Conclusion

The present studies assessed various area characteristics and different biodiversity and landscape measures to relate them to the mental well-being of people on-site. They also connect the mental well-being of people on-site to an evaluation with an online study. In this, mental well-being did not depend on actual bird diversity or biotope types, but recalled restoration was positively influenced by perceived bird diversity. The perceived naturalness of an area positively influenced all mental well-being variables, while infrastructure and human impact in an area had a negative impact. Participants with a more positive perception of birds benefit in terms of mental well-being, possibly due to better connectedness to nature. Therefore, to increase the positive impact on the mental well-being of visitors to recreational areas, the perceived naturalness and perceived bird diversity of an area should be increased.

Perception of an area can also be retrieved by showing pictures and is not dependent on the experience on-site. More research needs to be done on the effect of mental well-being when looking at pictures. These results would be useful as a proxy for people who cannot easily access these areas, as well as for reducing the impact on these areas.

## Implications and limitations

Mental well-being depends mostly on the perception of the area, mainly perceived naturalness and perceived bird diversity, and is therefore indirectly connected to the infrastructure and human impact in an area. Positive perception of birds—and therefore probably a higher connectedness to nature—also benefits the mental well-being of participants. This has a plethora of implications for future studies and the planning of recreational areas in accordance with nature conservation. People express more positive emotions in places with less human impact, so recreational places should take this into account when planning, renovating, or advertising places. Coincidentally, places with less human impact are often areas where biodiversity thrives and are therefore important for nature conservation. Especially in cities, these natural and biodiverse places need to be established and preserved for the future, for the sake of nature and people’s mental well-being.

A few limitations need to be addressed on behalf of this study. Our results of people feeling better in areas with less human impact are statistically true for the study sites in this study. It may not be true for other areas, since the range of our study sites is quite limited to a small region inside of southwestern Germany. In other countries, this may be different not only due to ecological reasons but also due to cultural differences.

In addition, participants were people met by chance at the study sites. They were asked to participate after being in the study area. Therefore our sample may be made up of people already happy to be outside and more inclined towards nature. But this was not controlled for here. Surveying the effect of being outside in the recreational areas on “city-people” or people with non-nature-related hobbies would be important to get a more complete picture.

The comparison of the online and outdoor studies should also be taken only as an indication since the online sample was mainly made up of university students and in its small sample size is not directly comparable to the outside study.

It would also be interesting to see how people on-site perceive the restorativeness of the areas. This was only asked in the picture survey and its impact therefore needs to be studied further.

Amidst the ever-growing human impact, we think that our results of the impact of perception/experience are an important factor to consider when designing recreational areas in the future. To be able to boost mental well-being and positive experiences in outdoor spaces, these areas should be designed to seem natural to recreationists, but still manageable to facilitate usage.

## Electronic supplementary material

Below is the link to the electronic supplementary material.

Supplementary Material 1

## Data Availability

The datasets analysed during the current study are available from the corresponding author on reasonable request.

## References

[1] International Health Conference. Constitution of the world health organization. *Bull. World Health Org.* **80**(12), 983–984 (2002). 12571729 PMC2567705

[2] Montgomery, M. United Nations Population Fund: State of world population 2007: Unleashing the potential of urban growth. *Popul. Dev. Rev.* **33**(3), 639–641 (2007).

[3] United Nations, Department of Economic and Social Affairs, Population Division (2018). World Urbanization Prospects: The 2018 Revision, Online Edition.

[4] McDonald, R. I. et al. Research gaps in knowledge of the impact of urban growth on biodiversity. *Nat. Sustain.* **3**(1), 16–24 (2020).

[5] McDonald, R. I., Marcotullio, P. J. & Güneralp, B. Urbanization and global trends in biodiversity and ecosystem services. In *Urbanization, biodiversity and ecosystem services: challenges and opportunities: a global assessment* (eds Elmqvist, T. et al.) 31–52 (Springer, 2013).

[6] Houlden, V., Jani, A. & Hong, A. Is biodiversity of greenspace important for human health and wellbeing? A bibliometric analysis and systematic literature review. *Urban For. Urban Green.* **66**, 127385 (2021).

[7] Aerts, R., Honnay, O. & Van Nieuwenhuyse, A. Biodiversity and human health: Mechanisms and evidence of the positive health effects of diversity in nature and green spaces. *Br. Med. Bull.* **127**(1), 5–22 (2018). 30007287 10.1093/bmb/ldy021

[8] Lovell, R., Wheeler, B. W., Higgins, S. L., Irvine, K. N. & Depledge, M. H. A systematic review of the health and well-being benefits of biodiverse environments. *J. Toxicol. Environ. Health Part B* **17**(1), 1–20 (2014). 10.1080/10937404.2013.856361 24597907

[9] Linton, M. J., Dieppe, P. & Medina-Lara, A. Review of 99 self-report measures for assessing well-being in adults: Exploring dimensions of well-being and developments over time. *BMJ Open* **6**(7), e010641 (2016). 27388349 10.1136/bmjopen-2015-010641 PMC4947747

[10] Korpela, K., Pasanen, T. & Ratcliffe, E. *Biodiversity and psychological well-being. Urban biodiversity* 134–149 (Routledge, 2018).

[11] Marselle, M. R., Martens, D., Dallimer, M. & Irvine, K. N. Review of the mental health and well-being benefits of biodiversity. In *Biodiversity and Health in the Face of Climate Change* (eds Marselle, M. R. et al.) 175–211 (Springer, 2019).

[12] White, M. P., Alcock, I., Wheeler, B. W. & Depledge, M. H. Would you be happier living in a greener urban area? A fixed-effects analysis of panel data. *Psychol. Sci.* **24**(6), 920–928 (2013). 23613211 10.1177/0956797612464659

[13] Beyer, K. M. et al. Exposure to neighborhood green space and mental health: Evidence from the survey of the health of Wisconsin. *Int. J. Environ. Res. Public Health* **11**(3), 3453–3472 (2014). 24662966 10.3390/ijerph110303453 PMC3987044

[14] Wei, H. et al. The association between plant diversity and perceived emotions for visitors in urban forests: A pilot study across 49 parks in China. *Urban For. Urban Green.* **73**, 127613 (2022).

[15] Kaplan, R. & Kaplan, S. *The Experience of Nature: A Psychological Perspective* (Cambridge University Press, 1989).

[16] Kaplan, S. The restorative benefits of nature: Toward an integrative framework. *J. Environ. Psychol.* **15**(3), 169–182 (1995).

[17] Ulrich, R. S. Aesthetic and affective response to natural environment. In *Behavior and the Natural Environment* 85–125 (Springer, 1983).

[18] Ulrich, R. S. et al. Stress recovery during exposure to natural and urban environments. *J. Environ. Psychol.* **11**(3), 201–230 (1991).

[19] Marselle, M. R., Lindley, S. J., Cook, P. A. & Bonn, A. Biodiversity and health in the urban environment. *Curr. Environ. Health Rep.* **8**(2), 146–156 (2021). 33982150 10.1007/s40572-021-00313-9 PMC8115992

[20] Collins, R. M. et al. A systematic map of research exploring the effect of greenspace on mental health. *Landsc. Urban Plan.* **201**, 103823 (2020).

[21] Sandifer, P. A., Sutton-Grier, A. E. & Ward, B. P. Exploring connections among nature, biodiversity, ecosystem services, and human health and well-being: Opportunities to enhance health and biodiversity conservation. *Ecosyst. Serv.* **12**, 1–15 (2015).

[22] Cox, D. T. et al. Doses of neighbourhood nature: The benefits for mental health of living with nature. *BioScience* **67**(2), 147–155 (2017).

[23] Lindemann-Matthies, P., Junge, X. & Matthies, D. The influence of plant diversity on people’s perception and aesthetic appreciation of grassland vegetation. *Biol. Conserv.* **143**(1), 195–202 (2010).

[24] Qiu, L., Lindberg, S. & Nielsen, A. B. Is biodiversity attractive?—On-site perception of recreational and biodiversity values in urban green space. *Landsc. Urban Plan.* **119**, 136–146 (2013).

[25] Lindemann-Matthies, P. & Matthies, D. The influence of plant species richness on stress recovery of humans. *Web Ecol.* **18**(2), 121–128 (2018).

[26] Methorst, J., Bonn, A., Marselle, M., Böhning-Gaese, K. & Rehdanz, K. Species richness is positively related to mental health—A study for Germany. *Landsc. Urban Plan.* **211**, 104084. 10.1016/j.landurbplan.2021.104084 (2021).

[27] Cameron, R. W. F. et al. Where the wild things are! Do urban green spaces with greater avian biodiversity promote more positive emotions in humans?. *Urban Ecosyst.* **23**(2), 301–317. 10.1007/s11252-020-00929-z (2020).

[28] Butler, S. J., Boccaccio, L., Gregory, R. D., Vorisek, P. & Norris, K. Quantifying the impact of land-use change to European farmland bird populations. *Agric. Ecosyst. Environ.* **137**(3–4), 348–357 (2010).

[29] Dallimer, M. et al. Biodiversity and the feel-good factor: Understanding associations between self-reported human well-being and species richness. *BioScience* **62**(1), 47–55 (2012).

[30] Wheeler, B. W. et al. Beyond greenspace: an ecological study of population general health and indicators of natural environment type and quality. *Int. J. Health Geogr.* **14**, 1–17 (2015). 25924685 10.1186/s12942-015-0009-5 PMC4455695

[31] Woods, B. Animals on display: Principles for interpreting captive wildlife. *J. Tour. Stud.* **9**(1), 28–39 (1998).

[32] Vanhöfen, J., Schöffski, N., Härtel, T. & Randler, C. Are lay people able to estimate breeding bird diversity?. *Animals* **12**(22), 3095 (2022). 36428323 10.3390/ani12223095 PMC9686614

[33] Hooykaas, M. J. et al. Identification skills in biodiversity professionals and laypeople: A gap in species literacy. *Biol. Conserv.* **238**, 108202 (2019).

[34] Parra-Saldívar, A., Abades, S., Celis-Diez, J. L. & Gelcich, S. Exploring perceived well-being from urban parks: Insights from a megacity in Latin America. *Sustainability* **12**(18), 7586 (2020).

[35] Shwartz, A., Tzunz, M., Gafter, L. & Colléony, A. One size does not fit all: The complex relationship between biodiversity and psychological well-being. *Urban For. Urban Green.* **86**, 128008. 10.1016/j.ufug.2023.128008 (2023).

[36] Boley, B. B. et al. Measuring place attachment with the abbreviated place attachment scale (APAS). *J. Environ. Psychol.* **74**, 101577 (2021).

[37] Bryce, R. et al. Subjective well-being indicators for large-scale assessment of cultural ecosystem services. *Ecosyst. Serv.* **21**, 258–269. 10.1016/j.ecoser.2016.07.015 (2016).

[38] Droseltis, O. & Vignoles, V. L. Towards an integrative model of place identification: Dimensionality and predictors of intrapersonal-level place preferences. *J. Environ. Psychol.* **30**(1), 23–34 (2010).

[39] Lewicka, M. Place attachment: How far have we come in the last 40 years?. *J. Environ. Psychol.* **31**(3), 207–230 (2011).

[40] Randler, C., Friedrich, S. & Koch, S. Psychological restoration, place attachment and satisfaction in birders and non-birding visitors. *J. Outdoor Recreat. Tour.* **44**, 100679 (2023).

[41] Scannell, L. & Gifford, R. Defining place attachment: A tripartite organizing framework. *J. Environ. Psychol.* **30**(1), 1–10 (2010).

[42] Subiza-Pérez, M., Vozmediano, L. & San Juan, C. Welcome to your plaza: Assessing the restorative potential of urban squares through survey and objective evaluation methods. *Cities* **100**, 102461 (2020).

[43] Menatti, L., Subiza-Pérez, M., Villalpando-Flores, A., Vozmediano, L. & San Juan, C. Place attachment and identification as predictors of expected landscape restorativeness. *J. Environ. Psychol.* **63**, 36–43 (2019).

[44] Young, C., Hofmann, M., Frey, D., Moretti, M. & Bauer, N. Psychological restoration in urban gardens related to garden type, biodiversity and garden-related stress. *Landsc. Urban Plan.* **198**, 103777. 10.1016/j.landurbplan.2020.103777 (2020).

[45] Hepburn, L., Smith, A. C., Zelenski, J. & Fahrig, L. Bird diversity unconsciously increases people’s satisfaction with where they live. *Land* **10**(2), 153 (2021).

[46] Nghiem, T. et al. Biodiverse urban forests, happy people: Experimental evidence linking perceived biodiversity, restoration, and emotional wellbeing. *Urban For. Urban Green.* **59**, 127030 (2021).

[47] Carrus, G. et al. Go greener, feel better? The positive effects of biodiversity on the well-being of individuals visiting urban and peri-urban green areas. *Landsc. Urban Plan.* **134**, 221–228 (2015).

[48] Chang, J., Wu, C.-C. & Chang, C.-Y. Landscape naturalness and restoring benefit: A connection through bird diversity. *Urban Ecosyst.* **27**(1), 41–50. 10.1007/s11252-023-01425-w (2024).

[49] Schebella, M. F., Weber, D., Schultz, L. & Weinstein, P. The wellbeing benefits associated with perceived and measured biodiversity in Australian urban green spaces. *Sustainability* **11**(3), 802 (2019).

[50] World Health Organization. Regional Office for, E. (2016). *Urban green spaces and health*. https://iris.who.int/handle/10665/345751.

[51] White, M. P., Elliott, L. R., Gascon, M., Roberts, B. & Fleming, L. E. Blue space, health and well-being: A narrative overview and synthesis of potential benefits. *Environ. Res.* **191**, 110169. 10.1016/j.envres.2020.110169 (2020). 32971082 10.1016/j.envres.2020.110169

[52] Wyles, K. J. et al. Are Some natural environments more psychologically beneficial than others? The importance of type and quality on connectedness to nature and psychological restoration. *Environ. Behav.* **51**(2), 111–143. 10.1177/0013916517738312 (2019).

[53] Barton, J. & Pretty, J. What is the best dose of nature and green exercise for improving mental health? A multi-study analysis. *Environ. Sci. Technol.* **44**(10), 3947–3955 (2010). 20337470 10.1021/es903183r

[54] Grahn, P. & Stigsdotter, U. A. Landscape planning and stress. *Urban For. Urban Green.* **2**(1), 1–18 (2003).

[55] Hartig, T., Evans, G. W., Jamner, L. D., Davis, D. S. & Gärling, T. Tracking restoration in natural and urban field settings. *J. Environ. Psychol.* **23**(2), 109–123 (2003).

[56] Maas, J. et al. Morbidity is related to a green living environment. *J. Epidemiol. Community Health* **63**(12), 967–973 (2009). 19833605 10.1136/jech.2008.079038

[57] Maas, J., Verheij, R. A., Groenewegen, P. P., De Vries, S. & Spreeuwenberg, P. Green space, urbanity, and health: How strong is the relation?. *J. Epidemiol. Community Health* **60**(7), 587–592 (2006). 16790830 10.1136/jech.2005.043125 PMC2566234

[58] Ottosson, J. & Grahn, P. A comparison of leisure time spent in a garden with leisure time spent indoors: On measures of restoration in residents in geriatric care. *Landsc. Res.* **30**(1), 23–55 (2005).

[59] Roe, J. & Aspinall, P. The restorative benefits of walking in urban and rural settings in adults with good and poor mental health. *Health Place* **17**(1), 103–113 (2011). 21094074 10.1016/j.healthplace.2010.09.003

[60] Subiza-Pérez, M. et al. Exploring psychological restoration in favorite indoor and outdoor urban places using a top-down perspective. *J. Environ. Psychol.* **78**, 101706 (2021).

[61] Venter, O. et al. Sixteen years of change in the global terrestrial human footprint and implications for biodiversity conservation. *Nat. Commun.*10.1038/ncomms12558 (2016). 27552116 10.1038/ncomms12558 PMC4996975

[62] Stein, A., Gerstner, K. & Kreft, H. Environmental heterogeneity as a universal driver of species richness across taxa, biomes and spatial scales. *Ecol. Lett.* **17**(7), 866–880 (2014). 24751205 10.1111/ele.12277

[63] Fuller, R. A., Irvine, K. N., Davies, Z. G., Armsworth, P. R., & Gaston, K. J. Interactions between people and birds in urban landscapes. (2013).

[64] Shwartz, A., Turbé, A., Simon, L. & Julliard, R. Enhancing urban biodiversity and its influence on city-dwellers: An experiment. *Biol. Conserv.* **171**, 82–90 (2014).

[65] Schebella, M., Weber, D., Schultz, L. & Weinstein, P. In pursuit of urban sustainability: Predicting public perceptions of park biodiversity using simple assessment tools. *Int. J. Environ. Res.* **13**(4), 707–720 (2019).

[66] Gidlow, C. et al. Development of the natural environment scoring tool (NEST). *Urban For. Urban Green.* **29**, 322–333 (2018).

[67] Voigt, A., Kabisch, N., Wurster, D., Haase, D. & Breuste, J. Structural diversity: A multi-dimensional approach to assess recreational services in urban parks. *Ambio* **43**(4), 480–491 (2014). 24740619 10.1007/s13280-014-0508-9 PMC3989521

[68] Meng, L., Li, S. & Zhang, X. Exploring biodiversity’s impact on mental well-being through the social-ecological lens: Emphasizing the role of biodiversity characteristics and nature relatedness. *Environ. Impact Assess. Rev.* **105**, 107454. 10.1016/j.eiar.2024.107454 (2024).

[69] Lee, J. H. & Scott, D. Measuring birding specialization: A confirmatory factor analysis. *Leisure Sci.* **26**(3), 245–260 (2004).

[70] Randler, C. & Heil, F. Determinants of bird species literacy—Activity/interest and specialization are more important than socio-demographic variables. *Animals* **11**(6), 1595 (2021). 34071521 10.3390/ani11061595 PMC8229662

[71] Randler, C. An analysis of heterogeneity in German speaking birdwatchers reveals three distinct clusters and gender differences. *Birds* **2**(3), 250–260 (2021).

[72] Belaire, J. A., Westphal, L. M., Whelan, C. J. & Minor, E. S. Urban residents’ perceptions of birds in the neighborhood: Biodiversity, cultural ecosystem services, and disservices. *Condor Ornithol. Appl.* **117**(2), 192–202 (2015).

[73] Ferraro, D. M. et al. The phantom chorus: Birdsong boosts human well-being in protected areas. *Proc. R. Soc. B Biol. Sci.* **287**(1941), 20201811. 10.1098/rspb.2020.1811 (2020). 10.1098/rspb.2020.1811 PMC7779501 33323075

[74] Southon, G. E., Jorgensen, A., Dunnett, N., Hoyle, H. & Evans, K. L. Perceived species-richness in urban green spaces: Cues, accuracy and well-being impacts. *Landsc. Urban Plan.* **172**, 1–10. 10.1016/j.landurbplan.2017.12.002 (2018).

[75] Bibby, C. J., Burgess, N. D., Ecologists, E. A., Hillis, D. M., Hill, D. A., Ornithology, B. T. F., Mustoe, S., Birds, R. S. F. T. P. O., Lambton, S., & International, B. *Bird Census Techniques*. (Elsevier Science, 2000). https://books.google.de/books?id=Ld5wkzPp49cC

[76] Höll, N., Gerstner, H., Raddatz, J., Murmann-Kristen, L., Mast, R., Breunig, T., & Demuth, S. Arten, Biotope, Landschaft. *Schlüssel zum Erfassen, Beschreiben, Bewerten. fourth ed. LUBW, Karlsruhe*, (2009).

[77] Reichert, G. Interrelationships between humans and birds: How urbanization affects the ecape behaviour of birds and possible consequences in the Feel-Good Factor of nature space visitors. Eberhard Karls Universität Tübingen. (2022). [Unpublished Bachelor Thesis]

[78] Bundesamt für Kartographie und Geodäsie. (2023). Produkte und Dienste. Assessed 30 Aug, 2024; https://gdz.bkg.bund.de.

[79] Grellier, J. et al. BlueHealth: A study programme protocol for mapping and quantifying the potential benefits to public health and well-being from Europe’s blue spaces. *BMJ Open* **7**(6), e016188 (2017). 28615276 10.1136/bmjopen-2017-016188 PMC5726080

[80] Venter, O., Sanderson, E. W., Magrach, A., Allan, J. R., Beher, J., Jones, K. R., Possingham, H. P., Laurance, W. F., Wood, P., Fekete, B. M., Levy, M. A., & Watson, J. E. *Last of the Wild Project, Version 3 (LWP-3): 2009 Human Footprint, 2018 Release* NASA Socioeconomic Data and Applications Center (SEDAC). 10.7927/H46T0JQ4 (2018).

[81] White, M. P. et al. Marine wildlife as an important component of coastal visits: The role of perceived biodiversity and species behaviour. *Marine Policy* **78**, 80–89 (2017).

[82] Fuller, R. A., Irvine, K. N., Devine-Wright, P., Warren, P. H. & Gaston, K. J. Psychological benefits of greenspace increase with biodiversity. *Biol. Lett.* **3**(4), 390–394 (2007). 17504734 10.1098/rsbl.2007.0149 PMC2390667

[83] Feng, X. et al. Green space quality and adolescent mental health: Do personality traits matter?. *Environ. Res.* **206**, 112591 (2022). 34932980 10.1016/j.envres.2021.112591

[84] Mayer, F. S. & Frantz, C. M. The connectedness to nature scale: A measure of individuals’ feeling in community with nature. *J. Environ. Psychol.* **24**(4), 503–515. 10.1016/j.jenvp.2004.10.001 (2004).

[85] Cheng, J. C. H. & Monroe, M. C. Connection to nature: Children’s affective attitude toward nature. *Environ. Behav.* **44**(1), 31–49 (2012).

[86] Taylor, L., Hahs, A. K. & Hochuli, D. F. Wellbeing and urban living: Nurtured by nature. *Urban Ecosyst.* **21**, 197–208 (2018).

[87] Cox, D. T. & Gaston, K. J. Likeability of garden birds: Importance of species knowledge & richness in connecting people to nature. *PloS one* **10**(11), e0141505 (2015). 26560968 10.1371/journal.pone.0141505 PMC4641628

[88] Methorst, J. et al. The importance of species diversity for human well-being in Europe. *Ecol. Econ.* **181**, 106917. 10.1016/j.ecolecon.2020.106917 (2021).

[89] Ward Thompson, C. *Landscape and Health Special Issue* Vol. 41, 591–597 (Taylor & Francis, 2016).

[90] Li, J. et al. Comparative study of the physiological and psychological effects of forest and urban auditory stimulus on humans. *Int. J. Geoherit. Parks* **9**(3), 363–373. 10.1016/j.ijgeop.2021.09.001 (2021).

[91] Hartig, T., Mitchell, R., De Vries, S. & Frumkin, H. Nature and health. *Ann. Rev. Public Health* **35**, 207–228 (2014). 24387090 10.1146/annurev-publhealth-032013-182443

[92] Martens, D., Gutscher, H. & Bauer, N. Walking in “wild” and “tended” urban forests: The impact on psychological well-being. *J. Environ. Psychol.* **31**(1), 36–44 (2011).

[93] Hussain, R. I. et al. Management of mountainous meadows associated with biodiversity attributes, perceived health benefits and cultural ecosystem services. *Sci. Rep.* **9**(1), 1497 (2019). 31628397 10.1038/s41598-019-51571-5 PMC6802121

[94] Gonçalves, P. et al. What’s biodiversity got to do with it? Perceptions of biodiversity and restorativeness in urban parks. *Ecol. Soc.*10.5751/ES-12598-260325 (2021).

[95] McEwan, K., Ferguson, F. J., Richardson, M. & Cameron, R. The good things in urban nature: A thematic framework for optimising urban planning for nature connectedness. *Landsc. Urban Plan.* **194**, 103687 (2020).

[96] Wilson, E. O. *Biophilia* (Harvard University Press, 1986).

[97] Wilson, E. O. Biophilia and the conservation ethic. In *Wilson Evolutionary Perspectives on Environmental Problems* (eds Dustin, J. P. & Iver Mysterud, E. O.) 250–258 (Routledge, 2017).

[98] Luo, S., Shi, J., Lu, T. & Furuya, K. Sit down and rest: Use of virtual reality to evaluate preferences and mental restoration in urban park pavilions. *Landsc. Urban Plan.* **220**, 104336 (2022).

