# Supplementary material for: The relationship between perception and landscape characteristics of recreational places with human mental well-being
Source: Sci Rep. 2025 Feb 4;15:4245. doi: 10.1038/s41598-025-88414-5 (PMC11794652; doi:10.1038/s41598-025-88414-5)
Supplement: Supplementary file 1 — Supplementary Material 1 [file 41598_2025_88414_MOESM1_ESM.pdf]

The relationship between perception and landscape characteristics of recreational places with human mental well-being.

Janina Vanhöfen*[a], Talia Härtel[a], Giovanna Reichert[a], Christoph Randler[a]

a: Didactics of Biology, Department of Biology, Eberhard Karls University Tübingen, Auf der Morgenstelle 24, D-72076 Tuebingen, Germany

* Correspondence: janina.vanhoefen@uni-tuebingen.de

JV: janina.vanhoefen@uni-tuebingen.de; https://orcid.org/0000-0001-7651-7346

TH: talia.haertel@uni-tuebingen.de; https://orcid.org/0000-0003-2074-5133

GR: giovanna.reichert99@gmail.com

CR: christoph.randler@uni-tuebingen.de; https://orcid.org/0000-0002-7357-2793

Table A1

Survey catalogue for the variables of the area perception on-site survey "Biodiversity and well-being" please note: this is the whole survey catalogue, the present manuscript does not analyze all of these items.

Concept	Items	Sources
**Reason for visit**	What was the main goal of your visit today?	Own
**Demographics**	Age<br>Gender<br>University degree	Well-established
**Birding specialization**	How many birds can you identify by Appearance?	Lee & Scott (2004), Randler & Heil (2021), Randler (2021)
**Place attachment - identity** (5-point Likert scale)	I feel very connected to this area here<br>This area is very special to me.<br>I can identify well with this area.	Boley et al. (2021)
**Place attachment - recovery** (5-point Likert scale)	Visiting this place gives me a feeling of freedom.<br>I can clear my head here.<br>Being here helps me to feel healthy.	Bryce et al. (2016)
**Loyalty** (5-point Likert scale)	I would also recommend this place to friends.	Prayag & Ryan (2012)
**Visitation frequency**	How often do you visit this area?	Prayag & Ryan (2012), Randler et al. (2007)
**Duration of visit**	How long did you spend in this area today?	Own
**Satisfaction** (5 categories)	Overall, how satisfied were you with your visit today?	Adapted from Arlinghaus (2006)
**Perceived bird diversity**	Please estimate: How many bird species are present in this area today?	Ferraro et al. (2020), Southon et al. (2018)
**Perceived naturalness** (5-point Likert scale)	How natural does this area seem to you?	Schebella et al. (2019)
**Perception of birds** (5-point Likert scale)	I like birds because they...<br>Are beautiful.<br>Have a nice song.<br>Make me Feel Good	Belaire et al. (2015)
**Recalled restoration**	How refreshed do you feel now compared to before your visit?	Young et al. (2020)
**Emotions** (5-point-Likert scale)	I feel…<br>Calm and relaxed<br>Peaceful<br>Refreshed	White et al. (2020), Wyles et al. (2019)
**Bird knowledge**	11 pictures of common bird species. Name the following bird species as accurately as possible.	Randler & Heil (2021), Randler et al. (2007), Dallimer et al. (2012)

Arlinghaus, R. (2006). On the apparently striking disconnect between motivation and satisfaction in recreational fishing: the case of catch orientation of German anglers. North American Journal of Fisheries Management, 26(3), 592-605.

Boley, B. B., Strzelecka, M., Yeager, E. P., Ribeiro, M. A., Aleshinloye, K. D., Woosnam, K. M., & Mimbs, B. P. (2021). Measuring place attachment with the abbreviated place attachment scale (APAS). *Journal of Environmental Psychology*, *74*, 101577.

Bryce, R., Irvine, K. N., Church, A., Fish, R., Ranger, S., & Kenter, J. O. (2016). Subjective well-being indicators for large-scale assessment of cultural ecosystem services. *Ecosystem Services*, *21*, 258-269. https://doi.org/https://doi.org/10.1016/j.ecoser.2016.07.015.

Dallimer, M., Irvine, K. N., Skinner, A. M., Davies, Z. G., Rouquette, J. R., Maltby, L. L., Warren, P. H., Armsworth, P. R., & Gaston, K. J. (2012). Biodiversity and the feel-good factor: understanding associations between self-reported human well-being and species richness. *BioScience*, *62*(1), 47-55.

Ferraro, D. M., Miller, Z. D., Ferguson, L. A., Taff, B. D., Barber, J. R., Newman, P., & Francis, C. D. (2020). The phantom chorus: birdsong boosts human well-being in protected areas. *Proceedings of the Royal Society B: Biological Sciences*, *287*(1941), 20201811. https://doi.org/doi:10.1098/rspb.2020.1811

Lee, J. H., & Scott, D. (2004). Measuring birding specialization: A confirmatory factor analysis. *Leisure Sciences*, *26*(3), 245-260.

Prayag, G., & Ryan, C. (2012). Antecedents of tourists' loyalty to Mauritius: The role and influence of destination image, place attachment, personal involvement, and satisfaction. Journal of travel research, 51(3), 342-356

Randler, C., & Heil, F. (2021). Determinants of bird species literacy—activity/interest and specialization are more important than socio-demographic variables. *Animals*, 11(6), 1595.

Randler, C. (2021). An analysis of heterogeneity in German speaking birdwatchers reveals three distinct clusters and gender differences. Birds, 2(3), 250-260.

Randler, C., Höllwarth, A., & Schaal, S. (2007). Urban park visitors and their knowledge of animal species. *Anthrozoös*, 20(1), 65-74.

Southon, G. E., Jorgensen, A., Dunnett, N., Hoyle, H., & Evans, K. L. (2018). Perceived species-richness in urban green spaces: Cues, accuracy and well-being impacts. *Landscape and Urban Planning*, *172*, 1-10. https://doi.org/https://doi.org/10.1016/j.landurbplan.2017.12.002

Schebella, M., Weber, D., Schultz, L., & Weinstein, P. (2019a). In Pursuit of Urban Sustainability: Predicting public perceptions of park biodiversity using simple assessment tools. *International Journal of Environmental Research*, *13*(4), 707-720.

Schebella, M. F., Weber, D., Schultz, L., & Weinstein, P. (2019b). The wellbeing benefits associated with perceived and measured biodiversity in Australian urban green spaces. *Sustainability*, *11*(3), 802.

Young, C., Hofmann, M., Frey, D., Moretti, M., & Bauer, N. (2020). Psychological restoration in urban gardens related to garden type, biodiversity and garden-related stress. *Landscape and Urban Planning*, *198*, 103777. https://doi.org/https://doi.org/10.1016/j.landurbplan.2020.103777

White, M. P., Elliott, L. R., Gascon, M., Roberts, B., & Fleming, L. E. (2020). Blue space, health and well-being: A narrative overview and synthesis of potential benefits. *Environmental Research*, *191*, 110169. https://doi.org/https://doi.org/10.1016/j.envres.2020.110169

Wyles, K. J., White, M. P., Hattam, C., Pahl, S., King, H., & Austen, M. (2019). Are Some Natural Environments More Psychologically Beneficial Than Others? The Importance of Type and Quality on Connectedness to Nature and Psychological Restoration. *Environment and Behavior*, *51*(2), 111-143. https://doi.org/10.1177/0013916517738312

Table A2

Biotic elements of the field survey. In accordance with the determining key catalogue "Arten, Biotope, Landschaft" from the nature conservation administration Baden-Württemberg (LUBW) which serves as a uniform reference system for biological data collection projects (Höll et al., 2009).

Concept	Umbrella terms	Surveyed terms
**Bodies of water**	Streams/running waters	Near-natural section of stream
		Developed section of stream
		Semi-natural river section
		Developed river section
		Canal
		Ditch
	Still waters	Pond
		Semi-natural area of a lake, pond or pond
		Non-natural area of a still water body
		Small non-natural water body
**Special morphological forms of anthropogenic origin**	Hollow path	
	Dry stone wall	
**Meadows and pastures**	Wet meadow	
	Farm meadow of medium sites	Fatty meadow
		Lean meadow
	Meadow of medium sites	
	Intensive grassland or grassland seeding	
	Stepping plants population	
	Ornamental grassland	
**Submerged and floating-leaf vegetation, spring meadows, reedbeds and large sedge reeds**	Spring meadow	Small reedbeds
		Reedbeds
		Large sedge reed
**Fringing vegetation, dominant stands of tall herbaceous plants and ruderal vegetation**	Fringing vegetation of medium locations	
	Fringing vegetation in dry, warm locations	
	Dominant stands of tall herbaceous plants	
	Tall herbaceous meadow	
	Ruderal vegetation	
**Heaths, rough, sandy and dry grasslands**	Juniper heath	
	Nutrient-poor grassland	
	Dry grassland	
**Fields, special crops and field gardens**	Agricultural field	
	Perennial special crops	Vineyard
		Tree nursery
	Field garden	
**Woody areas and bushes**	Field copses and field hedges	Field copse
		Field hedge
	Bushes	Scrub of dry and warm locations
		Scrub of medium locations
	Shrubbery and hedges not native to the natural habitat or location	Natural or non-natural scrub
		Hedge of natural or non-natural habitat
		Hedge fence
	Trees	Alley or row of trees
		Group of trees
		Single tree
		Scattered fruit trees
		Structurally rich forest edge
**Forests**	Marsh, swamp and riparian forests	Riparian forest strip alongside watercourses

	Forests of dry and warm locations	Oak or hornbeam-oak forest in dry, warm locations
		Beech forest in dry, warm locations
		Pine forest in dry, warm locations
	Beech-rich forests of medium locations	Beech forest of alkaline sites
	Oak and hornbeam-oak forests of medium habitats	
	Coniferous forests	
	Successional forests	Successional forest of deciduous trees
		Successional forest of deciduous and coniferous trees
		Successional forest of conifers
	Non-natural forest stands	

Höll, N., Gerstner, H., Raddatz, J., Murmann-Kristen, L., Mast, R., Breunig, T., & Demuth, S. (2009). Arten, Biotope, Landschaft. *Schlüssel zum Erfassen, Beschreiben, Bewerten. fourth ed. LUBW, Karlsruhe.*

Table A3

Abiotic elements used in the area assessment survey.

Source	Concept/Category	Umbrella terms	Surveyed terms
(Höll et al., 2009)	**Buildings**	Areas covered by buildings	Houses/buildings
			Industry
		Observation towers	
		Track areas	
Categories in adherence to Gidlow et al. (2018) and Schebella et al. (2019)	**Structural Elements**	Roads/paths	Concrete/Asphalt
			Paving
			Gravel
			Field paths/unpaved paths
		Stairs	
		Bridges	
		Gardens	Leisure gardens
			Parks
	**Infrastructure**	Accessibility	Connection
			Inaccessible areas/spots
			Car parks
			Walking routes/paths
			Signposting
		Facilities	Benches
			Playground
			Barbecue area
			Toilets
			Lighting
			Kiosk/restaurant/vending machines
			Roofing
			Waste bins, also for dogs
	**Incivilities**	Incivilities	Rubbish
			Graffiti
			Vandalism
			Dog faeces

Gidlow, C., van Kempen, E., Smith, G., Triguero-Mas, M., Kruize, H., Gražulevičienė, R., Ellis, N., Hurst, G., Masterson, D., & Cirach, M. (2018). Development of the natural environment scoring tool (NEST). *Urban Forestry & Urban Greening*, *29*, 322-333.

Höll, N., Gerstner, H., Raddatz, J., Murmann-Kristen, L., Mast, R., Breunig, T., & Demuth, S. (2009). Arten, Biotope, Landschaft. *Schlüssel zum Erfassen, Beschreiben, Bewerten. fourth ed. LUBW, Karlsruhe.*

Schebella, M., Weber, D., Schultz, L., & Weinstein, P. (2019). In Pursuit of Urban Sustainability: Predicting public perceptions of park biodiversity using simple assessment tools. *International Journal of Environmental Research*, *13*(4), 707-720.

Table A4

Survey catalogue for variables of the area perception online-study "Beauty of recreational areas"

Concept	Items	Source
**Demographics**	Age<br>Gender<br>Highest degree	
**Closeness to nature**<br>(5 point likert scale)	How often do you go for walks in nature?	Randler & Heil (2021)
**Area Assessment:**<br>4 pictures per locations and the following questions (5-point Likert scales):		
**Beauty**	How beautiful do you find this place?	Own
**Naturalness**	How natural does this area seem to you?	Schebella et al. (2019)
**Estimated Biodiversity**	Would you say that this area has low, medium or high biodiversity compared to others in the Rottenburg am Neckar/Stuttgart area? (Species diversity means the number of animals and plants)	White et al. (2017)
**Estimated restorativeness**	How well would a visit to this place help you to feel refreshed and revitalised?	White et al. (2017)

Randler, C., & Heil, F. (2021). Determinants of bird species literacy—activity/interest and specialization are more important than socio-demographic variables. *Animals*, 11(6), 1595.

Schebella, M., Weber, D., Schultz, L., & Weinstein, P. (2019). In Pursuit of Urban Sustainability: Predicting public perceptions of park biodiversity using simple assessment tools. *International Journal of Environmental Research*, *13*(4), 707-720

White, M. P., Weeks, A., Hooper, T., Bleakley, L., Cracknell, D., Lovell, R., & Jefferson, R. L. (2017). Marine wildlife as an important component of coastal visits: the role of perceived biodiversity and species behaviour. *Marine Policy*, *78*, 80-89.

Table A5

Descriptive statistical information on the variables (N = 40).

		**Variable**	**Min**	**Max**	**Mean**	**SD**
Area Assessment on-site	Ecological data	Bird diversity	18	42	28.05	6.28
		Biotope types	7	22.5	13.4	3.87
	Landscape characteristics	Structures	2	10	5.51	1.83
		Infrastructure	1.5	11	6.46	2.19
		Incivilities	0	6	1.33	1.21
Area assessment off-site	Landscape characteristics	Urbanity	1	2	1.53	.51
		Area type	1	3	1.68	.57
		Human Footprint Index (HFI)	13	46	33.5	9.87
Pen-and-paper survey of recreationists on-site	Area perception on-site	Perceived bird diversity	2.55	4.77	3.55	.51
		Perceived naturalness	2.69	4.48	3.72	.39
	Mental well-being	Emotions	3.66	4.66	4.15	.22
		Recalled restoration	3.5	4.52	4.03	.25
		Place attachment-recovery	3.58	4.46	4.06	.214
		Place attachment - identity	2.64	4.33	3.66	.38
Online survey	Area perception online	Estimated beauty	2.56	4.61	3.65	.48
		Estimated naturalness	1.69	4.57	3.09	.77
		Estimated restoration value	2.36	4.49	3.49	.52
		Estimated biodiversity richness	1.94	4.43	3.2	.69
